# Aphthæ of the Neck of the Uterus

**Published:** 1842-11-26

**Authors:** 


*Aphthæ of the Neck of the Uterus.*—M. Conté lays down, in a memoir addressed to the academy, that aphthæ of the neck of the uterus, though not described by writers, is a very frequent affection.—*Prov. Med. Jour.*, Oct. 8, 1842.

